# Retinoic Acid Induces Functionally Suppressive Foxp3^+^RORγt^+^ T Cells *In Vitro*

**DOI:** 10.3389/fimmu.2021.675733

**Published:** 2021-08-10

**Authors:** Mónica Martínez-Blanco, Daniel Lozano-Ojalvo, Leticia Pérez-Rodríguez, Sara Benedé, Elena Molina, Rosina López-Fandiño

**Affiliations:** Food Allergy Group, Department of Bioactivity and Food Anaysis, Instituto de Investigación en Ciencias de la Alimentación (CIAL, CSIC-UAM), Madrid, Spain

**Keywords:** food allergy, regulatory T cells, Il-6, Il17, retinoic acid, Th17, suppressive, Foxp3^+^RORγt^+^ T cells

## Abstract

**Introduction:**

CD4^+^ T cells with regulatory function co-expressing Foxp3 and RORγt are linked to the development of oral tolerance towards innocuous food antigens in mice. This study aimed to discern the role played by IL-6 and retinoic acid (RA) in the *in vitro* generation of Foxp3^+^RORγt^+^ T cells and to investigate whether such cells have suppressive properties.

**Methods:**

CD4^+^CD25^-^ T cells isolated from the spleen of BALB/c mice, were stimulated in the presence of IL-2 alone or together with TFG-β and different concentrations of IL-6 and/or RA. Percentage of Foxp3^+^, RORγt^+^, IL-17^+^, Foxp3^+^RORγt^-^, Foxp3^+^RORγt^+^, and Foxp3^-^RORγt^+^ T cells within the total CD4^+^ T cell population, production of cytokines (IL-10 and IL-17A) and gene expression (*Foxp3, Rorc, Tgfb1, Il6, Il10*, and *Il17*) were assessed at different time points. The phenotype and ability of cells generated from CD4^+^CD44^-^CD62L^+^ cells in the presence of RA to suppress effector T cell proliferation was assessed.

**Results:**

TGF-β plus IL-6 induced the generation of Foxp3^+^ and double positive Foxp3^+^RORγt^+^ T cells to a higher extent than TGF-β alone at the beginning of the incubation period, although expression of Foxp3 subsequently declined. RA, added to TGF-β, increased *Foxp3* and *Rorc* expression and Foxp3 and RORγt transcription and promoted the differentiation of Foxp3^+^RORγt^-^ and Foxp3^+^RORγt^+^ cells that expressed and secreted IL-17. Foxp3^+^ T cells generated *in vitro* in presence of RA were functionally suppressive.

**Conclusions:**

Under the influence of IL-2 and TGF-β, suppressive Foxp3^+^RORγt^+^ T cells that express and secrete IL-17 can be produced *in vitro* and RA further contributes to stabilize this phenotype.

## Introduction

Intestinal homeostasis relies on the development of tolerance mechanisms that recognize symbiotic microorganisms and innocuous food antigens. In this respect, induced regulatory T (Treg) cells are considered essential for establishing peripheral tolerance by counteracting the activity of the different effector T helper (Th) cell subsets. Treg cells are usually described as CD4^+^ T cells expressing the IL-2 receptor alpha-chain (CD25) and the transcription factor forkhead box P3 (Foxp3), which is essential to their function and acts as a specific Treg cell marker, although, along with Foxp3^+^ Treg cells, CD4^+^ type 1 regulatory T (Tr1) cells represent a different subset of Treg cells defined by the expression of a number of other transcription factors, as well as IL-10, and lack expression of Foxp3 (1). In addition, double positive Foxp3^+^RORγt^+^ T cells with regulatory function against exacerbated Th2 responses have been found in the small intestinal and colonic lamina propria of mice, linked to a specific, but wide diversity of bacterial species (2–5). While the mechanisms underlying their generation are not fully clear, involvement of antigens and metabolites derived from the gut microbiota, which stimulate epithelial production of retinoic acid (RA), has been proposed (3, 6). In this respect, effective oral immunotherapy with peptides was also found to cause an increase in RORγt^+^ Treg cells through the enhancement of vitamin A metabolism (7, 8).

Foxp3^+^RORγt^+^ T cells generated *in vivo* were proved to be functionally suppressive, inhibiting *in vitro* proliferation of activated CD4^+^ effector T (Teff) cells and constraining inflammatory responses *in vivo*, and to constitute a distinct, stable cell lineage, rather than an intermediate subset of Treg and Th17 differentiation (2, 4, 9). Even if, according to some authors, the reciprocal generation of the transcription factors Foxp3 and RORγt precludes their co-existence *in vitro* (10–12), these double positive cells have been produced from mouse naïve cells under T cell receptor (TCR) stimulation in the presence of TGF-β (2, 13), although the factors implicated, and the functionality of the resulting cells have not been investigated in detail. In this study, we show that suppressive double positive Foxp3^+^RORγt^+^ T cells can be generated *in vitro* under the influence of IL-2 and TGF-β. Addition of pro-inflammatory cytokines is not a requisite for the simultaneous expression of Foxp3 and RORγt, but rather RA contributes to stabilize the phenotype of these double positive cells.

## Materials and Methods

### Animals

Female BALB/c mice (4-6 weeks of age) were from Charles River Laboratories (Saint Germain sur l´Arbresle, Rhône, France). All protocols involving animals followed the European legislation (Directive 2010/63/EU) and were approved by Comunidad de Madrid (Ref PROEX 286.8/20).

### Cell Isolation and *In Vitro* Generation of Putative Treg Cells

Spleens were physically disrupted through a 70 μm cell strainer (BD labware, New Jersey, US), washed to obtain single cell suspensions, and pooled (3-5 mice per pool) for further assays. CD4^+^ T cells were first isolated with a negative selection kit and CD4^+^CD25^-^ cells were sorted from CD4^+^ T cells using a CD25^+^ Treg positive selection kit. To obtain naïve CD4^+^ T cells (CD4^+^CD44^-^CD62L^+^), a negative selection kit was used. All kits were from StemCell Technologies (Vancouver, Canada).

Isolated T cells were cultured in RPMI 1640 medium supplemented with 10% FBS, 1% L-glutamine, 1% penicillin/streptomycin (Biowest SAS, Nuaillé, France), 100 mM sodium pyruvate, 50 μM β-mercaptoethanol, 10 mM HEPES, and 10% non-essential amino acids (Sigma-Aldrich, St. Louis, MO, USA) at a concentration of 1x10^6^ cells mL^-1^ with plate-bound anti-CD3 (10 μg mL^-1^, clone 17A2, eBioscience San Diego, USA) and soluble anti-CD28 (5 μg mL^-1^, clone 37.51, eBioscience) in the presence of IL-2 (20 ng mL^-1^, PeproTech, London, UK) for a maximum of 4 days. In some cases, TGF-β (5 ng mL^-1^, eBioscience), IL-6 (20 ng mL^-1^, eBioscience), and RA (0.01, 0.1, or 1 μM, Sigma-Aldrich) were also added to the culture. T cells were analyzed by flow cytometry and preserved for gene expression analyses.

### *In Vitro* Suppression Assays

To assess the suppressive function of induced putative Treg cells, CD4^+^ T cells were isolated using an EasySep negative selection kit (StemCell Technologies) and labelled with carboxyfluororescein succinimidyl ester (CFSE; CellTrace CFSE cell proliferation kit, Life Technologies, Carlsbad, CA, USA). Treg cells generated from CD4^+^ naïve T cells after 48 h and 96 h of stimulation were co-cultured with Teff cells (5x10^4^ cells mL^-1^) at different ratios (0:1, 1:2, 1:4, and 1:5) in a total volume of 200 µL of RPMI and in the presence of anti-CD3/28-coated latex beads (Molecular Probes Eugene, Oregon, USA) (5x10^4^ beads mL^-1^) for 72 h.

### Flow Cytometry of T Cells

After culture, cells were collected in PBS containing 2% FBS and 1mM EDTA. Live cells were determined with LIVE/DEAD^™^ Fixable Near-IR Dead Cell Stain Kit (Thermo Fisher Scientific). Fc receptors were blocked using anti-CD16/CD32 (clone 93, eBioscience) and samples were stained with anti-CD4-Alexa Fluor 700 (clone GK1.5), anti-Foxp3-PE (clone 150D/E4), anti-RORγt-APC (clone B2D), anti-IL17A-PerCP/Cy5.5 (clone eBio17B7), and anti-IL10-FITC (clone JES5-16E3) (all from eBioscience). Samples were fixed and permeabilized by using Foxp3 Transcription Factor Staining Buffer (eBiosience). At least 10^5^ cells were acquired with a Gallios flow cytometer and analyses were performed with Kaluza Analysis (version 1.3) (Beckman Coulter, Krefeld, Germany) and FlowJo softwares (version 10.6.2) (Ashland, OR, USA) following the gating strategy shown in **Supplementary Figure 1**.

### Gene Expression

RNA extraction and qPCR assays were performed as described (14). Primer pairs and thermal cycling conditions are included in **Supplementary Table 1**. Relative gene expression was calculated by normalizing data to the expression of the *Actb* gene (encoding for β-actin).

### Cytokine Analyses

The levels of IL-10 and IL-17A in cell culture supernatants were quantified by Luminex commercial kits following the manufacturer’s instructions (eBioscience).

### Statistical Analyses

All experiments were conducted at least in duplicate. Results are presented as means ± SEM. Differences between two experimental groups were assessed by the unpaired two-tailed Student’s t test. Differences among three or more conditions for the same time point were determined by one-way ANOVA, and differences between the same condition at different time points were evaluated by two-way ANOVA, both followed by Tukey *post-hoc* test. Gene expression data, that followed a non-parametrical distribution, which were evaluated by Mann-Whitney U test. p< 0.05 was considered statistically significant. Statistical analyses were performed using GraphPad Prism v5 (GraphPad Software Inc., San Diego, USA).

## Results

We initially attempted to generate double positive Foxp3^+^RORγt^+^ T cells *in vitro* and to identify the factors involved in their differentiation. Traditionally, Foxp3^+^ cells are induced from CD4^+^CD25^-^ cells by TCR triggering (by the addition of anti-CD3 and anti-CD28 to the culture) in the presence of TGF-β (15). TGF-β-dependent Foxp3^+^ cell generation is further promoted by IL-2, which also constrains the differentiation of the Th17 cell subset (16), and by RA (12). On the other hand, IL-6 combined with TGF-β opens the RORγt differentiation pathway, with additional RA favoring the generation of double positive cells (2). We, therefore, used TCR stimulation of CD4^+^CD25^-^ cells cultured in presence of IL-2, alone or together with TGF-β and its combinations with IL-6 and RA.

**Figure 1:**
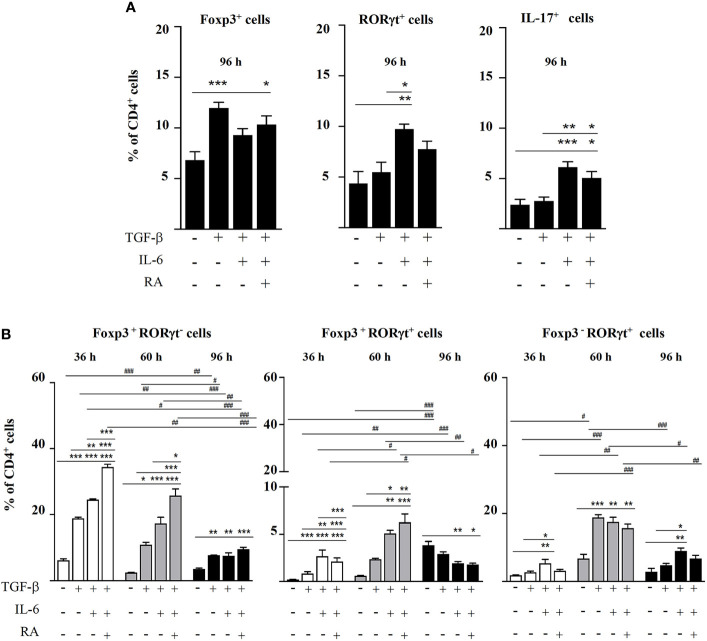
Magnetic sorted CD4^+^CD25^-^ spleen cells from BALB/c mice were stimulated with plate-bound anti-CD3 and soluble anti-CD28 in the presence of IL-2 (20 ng mL^-1^) and combinations of TGF-β (5 ng mL^-1^), IL-6 (20 ng mL^-1^), and RA (0.1 μM). **(A)** Percentage of Foxp3^+^, RORγt^+^, and IL-17^+^ cells within the total CD4^+^ T cell population after 96 h of culture. **(B)** Percentage of Foxp3^+^RORγt^-^, Foxp3^+^RORγt^+^, and Foxp3^-^RORγt^+^ T cells at different time points. Data are means of three different experiments (each performed with cells sorted from polled spleens from 3-5 mice) ± SEM (n= 3). *P < 0.05, **P < 0.01, ***P < 0.001 indicate statistically significant differences between culture conditions at each time point assessed by one-way ANOVA followed by Tukey *post-hoc *test, and ^#^ *P* < 0.05, ^##^ *P* < 0.01, ^###^ *P* < 0.001 indicate statistically significant differences between time points for each culture condition assessed by two-way ANOVA followed by Tukey *post-hoc *test.

As expected, following 96 h of culture, TGF-β induced the expansion of Foxp3^+^ cells from CD4^+^CD25^-^ cells, while TGF-β plus IL-6 increased the percentage of RORγt^+^ and IL-17^+^ cells, and TGF-β plus IL-6 and RA led to intermediate levels of these cells (**Figure 1A**). Most CD4^+^ cells were either Foxp3^+^RORγt^-^ or Foxp3^-^RORγt^+^, although a substantial proportion of cells expressed both Foxp3 and RORγt (**Figure 1B**). The study of the evolution of these cells over time showed that, following 36 and 60 h of culture, the combination of TGF-β with IL-6 and, particularly, with IL-6 and RA led to higher levels of Foxp3^+^RORγt^-^ and Foxp3^+^RORγt^+^ T cells, respectively, than TGF-β alone, but the percentage of these cells subsequently declined throughout the incubation time, and after 96 h, it was similar to that found at basal conditions (**Figure 1B**). This shows that IL-6 is able to drive co-expression of Foxp3 and RORγt, and that RA contributes to expand its impact on the population of Foxp3^+^ cells and double positive cells, although the effect is transient.

**Figure 2:**
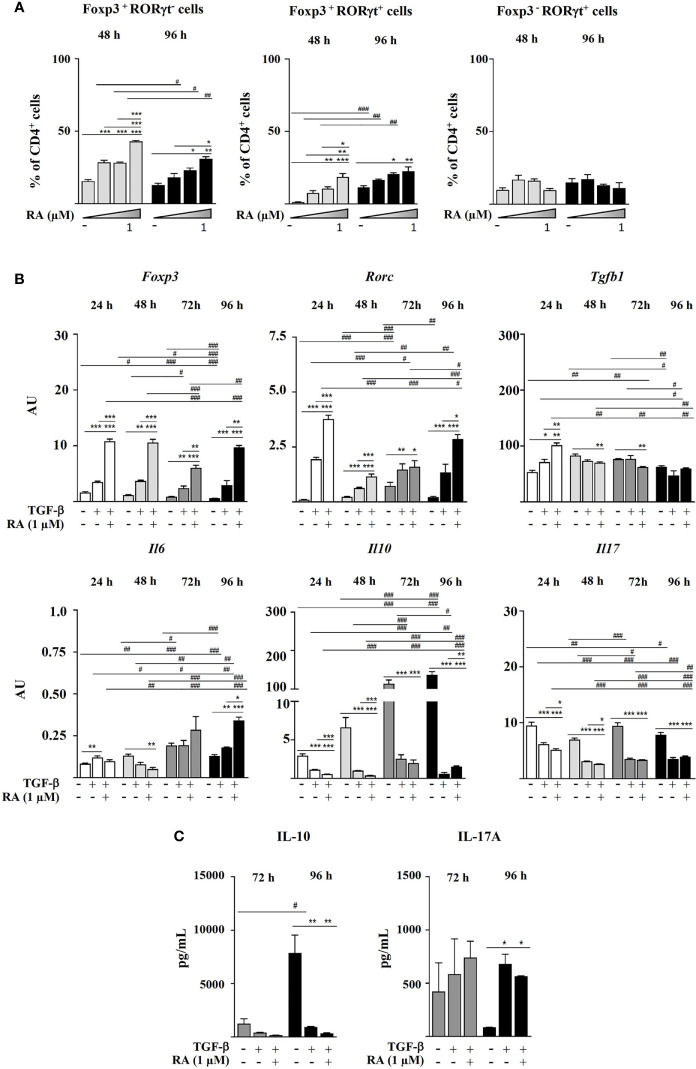
Magnetic sorted CD4^+^CD25^-^ spleen cells from BALB/c mice were stimulated with plate-bound anti-CD3 and soluble anti-CD28 in the presence of IL-2 (20 ng mL^-1^), TGF-β (5 ng mL^-1^), and RA (0.01, 0.1 or 1.0 μM). **(A)** Percentage of Foxp3^+^, RORγt^+^, Foxp3^+^RORγt^-^, Foxp3^+^RORγt^+^, and Foxp3^-^RORγt^+^ cells within the total CD4^+^ T cell population at different time points. **(B)** Gene expression of *Foxp3, Rorc, Tgfb1, Il6, Il10*, and *Il17*, normalized to the reference gene *Actb* and expressed in arbitrary units (AU). **(C)** Secretion of IL-10 and IL-17A. Data are means of three different experiments (each performed with cells sorted from polled spleens from 3-5 mice) ± SEM (n = 3). *P < 0.05, **P < 0.01, ***P < 0.001 indicate statistically significant differences between culture conditions at each time point assessed by one-way ANOVA followed by Tukey *post-hoc *test, and ^#^ *P* < 0.05, ^##^ *P* < 0.01, ^###^ *P* < 0.001 indicate statistically significant differences between time points for each culture condition assessed by two-way ANOVA followed by Tukey *post-hoc *test. For gene expression data statistical differences were evaluated by Mann-Whitney U test.

Next, we decided to assess the influence of different RA concentrations in the absence of IL-6 on the generation of Foxp3^+^RORγt^+^ T cells. The results showed that RA dose-dependently increased Foxp3 expression, which peaked at 24 h and remained fairly stable afterwards, and it also increased the expression of RORγt, although, in this case, a dose-response effect was not observed (not shown). Consequently, after 96 h, the cells treated with RA maintained increased levels of Foxp3 and RORγt expression, with a predominance of Foxp3^+^RORγt^-^ and Foxp3^+^RORγt^+^ cells over Foxp3^-^RORγt^+^ cells (**Figure 2A**). Therefore, *in vitro* culture with TGF-β in the presence of pro-inflammatory cytokines, such as IL-6, was not a requisite for CD4^+^CD25^-^ cells to simultaneously express Foxp3 and RORγt, but rather RA contributed to expand and stabilize the phenotype of double positive cells induced by TGF-β.

The study of the evolution of gene expression showed that TGF-β upregulated *Foxp3*, *Rorc*, and *Tgfb1* after 24 h of culture, particularly when combined with RA. *Rorc* expression subsequently declined to increase again at 72-96 h, probably linked to the upregulation of *Il6* (**Figure 2B**). Addition of both TGF-β and TGF-β plus RA to CD4^+^CD25^-^ cells downregulated *Il10* and *Il17* expression as compared with TCR stimulation in the presence of IL-2 alone (**Figure 2B**). However, while this caused a decline in IL-10 secretion, cells treated with TGF-β and TGF-β plus RA produced the highest IL-17A levels following 96 h of culture (**Figure 2C**).

**Figure 3:**
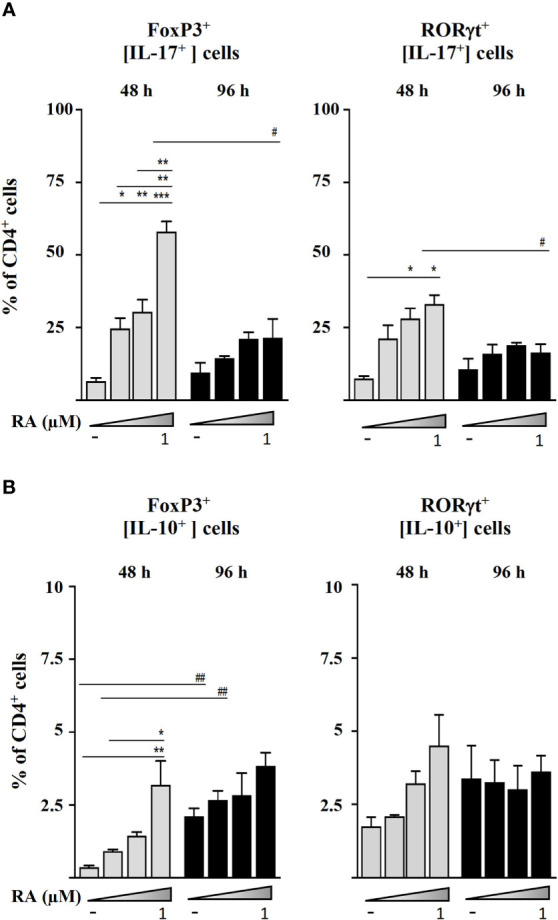
Magnetic sorted CD4^+^CD25^-^ spleen cells from BALB/c mice were stimulated with plate-bound anti-CD3 and soluble anti-CD28 in the presence of IL-2 (20 ng mL^-1^), TGF-β (5 ng mL^-1^), and RA (0.01, 0.1, or 1.0 μM). **(A)** Intracellular IL-17 expression in Foxp3^+^ and RORγt^+^ cells after 48 and 96 h of culture. **(B)** Intracellular IL-10 expression in Foxp3^+^ and RORγt^+^ cells after 48 and 96 h of culture. Data are means of three different experiments (each performed with cells sorted from polled spleens from 3-5 mice) ± SEM (n = 3). *P < 0.05, **P < 0.01, ***P < 0.001 indicate statistically significant differences between culture conditions at each time point assessed by one-way ANOVA followed by Tukey *post-hoc *test, and ^#^ *P* < 0.05, ^##^ *P* < 0.01 indicate statistically significant differences between time points for each culture condition assessed by two-way ANOVA followed by Tukey *post-hoc* test.

In order to investigate the cellular origin of IL-17 and IL-10 that accumulated in culture supernatants, we studied the intracellular expression of these cytokines by the different CD4^+^ cell populations (**Figure 3**). The results supported that RA induced the secretion of IL-17 by Foxp3^+^ and RORγt^+^ T cells in a dose-response manner after 48 h of culture (**Figure 3A**). However, as shown in **Figure 3B**, RA promoted a much lower IL-10 expression by Foxp3^+^ T cells after 48 h and only at the highest concentration assayed (1 µM).

**Figure 4:**
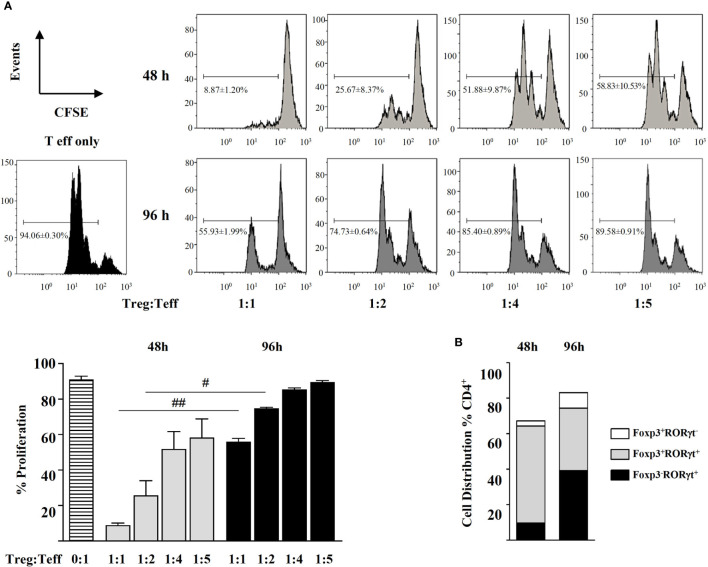
Magnetic sorted naïve CD4^+^CD44^-^CD62L^+^ spleen cells from BALB/c mice were stimulated with plate-bound anti-CD3 and soluble anti-CD28 in the presence of IL-2 (20 ng mL^-1^), TGF-β (5 ng mL^-1^), and RA (0.1 μM) for 48 and 96 h. **(A)** Suppressive activity of the cells thus generated (Treg) in co-cultures with different ratios of CFSE-labelled CD4^+^ effector T cells (Teff) in the presence of anti-CD3- and anti-CD28-coated latex beads for 72h. **(B)** Distribution of the generated Foxp3^+^RORγt^-^, Foxp3^+^RORγt^+^, and Foxp3^-^RORγt^+^ T cells after 48 and 96 h of culture. Data are means of three different experiments (each performed with cells sorted from polled spleens from 5 mice) ± SEM (n = 3). ^#^ *P* < 0.05, ^##^ *P* < 0.01 indicate statistically significant differences between time points for each culture condition assessed by the unpaired two-tailed Student’s t test.

In view that the classical conditions for *in vitro* generation Treg cells also led to Foxp3^+^RORγt^+^ T cells, we looked at the phenotype of CD44^-^CD62L^+^ naïve cells stimulated with TGF-β plus RA, known to be regulatory *in vitro* and *in vivo* (12). Indeed, after 48 h, these cells exerted a suppressive function in co-culture with different ratios of CD4^+^ Teff cells (**Figure 4A**). Flow cytometry analysis showed that they were largely Foxp3^+^RORγt^+^ cells (**Figure 4B**). After 96 h, even if the population of Foxp3^+^RORγt^+^ cells was maintained, there was an increase in the proportion of Foxp3^-^RORγt^+^ cells in the culture, which paralleled a decline in their ability to suppress the proliferation of CD4^+^ Teff cells (**Figures 4A, B**).

## Discussion

This study evidences the critical role played by RA in the *in vitro* generation and stabilization of Foxp3^+^RORγt^+^ T cell phenotype as well as the immunosuppressive ability of such cells. While Foxp3^+^RORγt^+^ cells had been previously induced *in vitro* from mouse naïve cells under TCR stimulation in the presence of TGF-β (2, 13), we initially used purified murine spleen CD4^+^ cells depleted of CD25^+^ cells that, in addition to CD44^-^CD62L^+^ naïve T cells, also contain CD44^+^CD62L^+^ central memory and CD44^+^CD62L^-^ effector memory cells, basically because naïve cells readily convert into Foxp3^+^ cells independently of factors relevant for Foxp3^+^ and RORγt^+^ cell generation, such as RA (17). In addition, CD44^+^CD62L^+^ cells can also be differentiated into Treg cells *in vitro* (18).

The observation that cells treated with TGF-β *in vitro* co-expressed the transcription factors Foxp3 and RORγt agrees with Lochner et al. (2), who reported that culture with TGF-β of TCR-stimulated naïve CD4^+^ T cells induces Foxp3^+^ Treg cells, all of which co-express RORγt after 72-96 h. Nevertheless, in contrast to studies showing that TGF-β in the presence of IL-6 preferentially generates pro-inflammatory Th17 cells from murine naïve CD4^+^ T cells *in vitro* (2, 10, 11), our results showed that, while TGF-β plus IL-6 enhanced the frequency of RORγt^+^ and IL-17^+^ cells with respect to TGF-β alone, IL-6 did not inhibit the conversion of CD4^+^CD25^-^ cells into Foxp3^+^ cells. In fact, TGF-β plus IL-6 induced the generation of Foxp3^+^ and double positive Foxp3^+^RORγt^+^ cells to a higher extent than TGF-β alone at the beginning of the incubation period, but expression of Foxp3 subsequently declined to levels similar to those of the untreated cells. Similarly, Zhou et al. (13) found that, following stimulation of naïve CD4^+^ T cells with anti-CD3, anti-CD28, and IL-2 in the presence of TGF-β plus IL-6, there was a substantial proportion of Foxp3^+^ cells, many of which also expressed IL-17. It should be noted that contradictory results have also been reported regarding the effect of IL-6 on the generation of Th17 or Treg cells *in vivo*. Thus, mice deficient for IL-6 have been described to develop either similar (2) or significantly less Foxp3^+^RORγt^+^ cells (3) than their IL-6 sufficient counterparts.

RA has been reported to drive the differentiation of a stable Treg cell lineage *in vitro* as compared with TGF-β alone and to counteract IL-6 activity through the blockade of IL-6 receptor expression, inhibiting the TGF-β plus IL-6-driven induction of Th17 cells (12). Accordingly, we found that RA, added to the combination of TGF-β and IL-6, promoted, following 60 h of culture, the generation of Foxp3^+^RORγt^-^ cells, but not that of Foxp3^-^RORγt^+^ T cells. However, RA also promoted the development of RORγt^+^ cells (19). Indeed, RA, added to TGF-β, increased *Foxp3* and *Rorc* expression and Foxp3 and RORγt transcription, and promoted the differentiation of Foxp3^+^RORγt^-^ and Foxp3^+^RORγt^+^ cells, even though, in agreement with previous findings (9), induction of RORγt was unstable, in view of the variable expression levels of *Rorc* mRNA.

TGF-β, either without or with RA, reduced the mRNA levels of the cytokines *Il10* and *Il17*, likely upregulated as a result of TCR stimulation of CD4^+^CD44^+^ memory T cells in the presence of IL-2. In fact, hindrance of cytokine secretion by CD44^+^ cells is one of the mechanisms through which RA favors the TGF-β-driven Foxp3 induction (17, 20). Nevertheless, we found that Foxp3^+^ T cells generated *in vitro* in presence of RA expressed and secreted IL-17, an observation which does not support the concept that induction of Foxp3 and IL-17 are mutually exclusive (13).

In order to evaluate the functionality of these cells, we attempted their generation from CD44^-^CD62L^+^ naïve T cells, which give rise to a higher proportion of Foxp3^+^ T cells and readily acquire suppressive properties *in vitro* as compared with memory T cells (17, 18). Noteworthy, most cells generated *in vitro* from naïve T cells with TGF-β and RA had, as expected, remarkable regulatory properties, but also a Foxp3^+^RORγt^+^ phenotype, suggesting that many studies that have referred to Treg cells induced by TGF-β and RA on naïve T cells as solely Foxp3^+^ cells did not discriminate between the two populations by appropriate staining for RORγt. While substantial differences may exist between the Foxp3^+^RORγt^+^ T cell subsets differentiated *in vitro* and *in vivo* (2), as well as between murine and human Treg cells (21), it should be noted that suppressive Foxp3^+^ cells that constitutively express RORγt and produce IL-17 upon activation can be found in human peripheral blood and lymphoid tissues (22, 23). IL-17 production may not correlate with the effector capacity of T cells, since simultaneous production of IL-10 could regulate the inflammatory response directing these cells to a regulatory function (24). However, the implication of IL-10 in the inhibition mediated by Foxp3^+^RORγt^+^ cells is controversial, with reports showing the preferential involvement of CTLA4 and IRF4, and even suggesting the participation of IL-17 in the control of exacerbated immune responses *in vivo* (3). The mechanisms of the regulatory action of Foxp3^+^RORγt^+^ T cells, as well as the role of RORγt and IL-17 deserve further investigations.

In conclusion, our results show that, under the influence of IL-2 and TGF-β, Foxp3^+^RORγt^+^ T cells that express and secrete IL-17 can be produced *in vitro*, and that RA further contributes to stabilize this phenotype, even if the conditions used for cell culture may conduct to cytokine production and to variations in the temporal regulation of the generation of double positive cells. Foxp3^+^RORγt^+^ T cells differentiated *in vitro* are able to prevent the proliferation of non-regulatory responder cells in co-culture experiments.

## Data Availability Statement

The raw data supporting the conclusions of this article will be made available by the authors, without undue reservation.

## Ethics Statement

The animal study was reviewed and approved by Comunidad de Madrid - PROEX 286.8/20.

## Author Contributions

MM-B, DL-O, and LP-R performed the experiments and interpreted the results. DL-O, SB, EM, and RL-F designed, planned, and supervised the study. RL-F wrote the manuscript and all authors revised it. All authors contributed to the article and approved the submitted version.

## Funding

This work was supported by Ministerio de Ciencia e Innovación through grants AGL2017-88964-R, FPU16/01974 (to LP-R) and JCI-2017-31345 (to SB).

## Conflict of Interest

The authors declare that the research was conducted in the absence of any commercial or financial relationships that could be construed as a potential conflict of interest.

## Publisher’s Note

All claims expressed in this article are solely those of the authors and do not necessarily represent those of their affiliated organizations, or those of the publisher, the editors and the reviewers. Any product that may be evaluated in this article, or claim that may be made by its manufacturer, is not guaranteed or endorsed by the publisher.

## References

[1] Noval Rivas M Chatila TA. Regulatory T Cells in Allergic Diseases. J Allergy Clin Immunol (2016) .138:639–52. 10.1016/j.jaci.2016.06.003 PMC5023156 27596705

[2] Lochner M Peduto L Cherrier M Sawa S Langa F Varona R. *In Vivo* Equilibrium of Proinflammatory IL-17+ and Regulatory IL-10+ Foxp3+ Rorγt+ T Cells. J Exp Med (2008) 205(6):1381–93. 10.1084/jem.20080034 PMC2413035 18504307

[3] Ohnmacht C Park JH Cording S Wing JB Atarashi K Obata Y. The Microbiota Regulates Type 2 Immunity Through Rorγt+ T Cells. Science (2015) .349(6251):989–93. 10.1126/science.aac4263 26160380

[4] Sefik E Geva-Zatorsky N Oh S Konnikova L Zemmour D McGuire AM. Individual Intestinal Symbionts Induce a Distinct Population of Rorγ+ Regulatory T Cells. Science (2015) 349(6251):993–7. 10.1126/science.aaa9420 PMC4700932 26272906

[5] Geva-Zatorsky N Sefik E Kua L Pasman L Tan TG Ortiz-Lopez A. Mining the Human Gut Microbiota for Immunomodulatory Organisms. Cell (2017) 168(5):928–43.e11. 10.1016/j.cell.2017.01.022 28215708 PMC7774263

[6] Schilderink R Verseijden C Seppen J Muncan V van den Brink GR Lambers TT. The SCFA Butyrate Stimulates the Epithelial Production of Retinoic Acid *via* Inhibition of Epithelial HDAC. Am J Physiol Gastrointest Liver Physiol (2016) 310(11):G1138–46. 10.1152/ajpgi.00411.2015 27151945

[7] Lozano-Ojalvo D Martínez-Blanco M Pérez-Rodríguez L Molina E Peláez C Requena T. Egg White Peptide-Based Immunotherapy Enhances Vitamin A Metabolism and Induces Rorγt+ Regulatory T Cells. J Fun Foods (2019) 52(1):204–11. 10.1016/j.jff.2018.11.012

[8] Lozano-Ojalvo D Martínez-Blanco M Pérez-Rodríguez L Molina E López-Fandiño R. Oral Immunotherapy With Egg Peptides Induces Innate and Adaptive Tolerogenic Responses. Mol Nutr Food Res (2019) 63(17):e1900144. 10.1002/mnfr.201900144 31140734

[9] Yang BH Hagemann S Mamareli P Lauer U Hoffmann U Beckstette M. Foxp3+ T Cells Expressing Rorγt Represent a Stable Regulatory T-Cell Effector Lineage With Enhanced Suppressive Capacity During Intestinal Inflammation. Mucosal Immunol (2016) 9(2):444–57. 10.1038/mi.2015.74 26307665

[10] Bettelli E Carrier Y Gao W Korn T Strom TB Oukka M. Reciprocal Developmental Pathways for the Generation of Pathogenic Effector TH17 and Regulatory T Cells. Nature (2006) 441(7090):235–8. 10.1038/nature04753 16648838

[11] Veldhoen M Hocking RJ Atkins CJ Locksley RM Stockinger B. Tgfβ in the Context of an Inflammatory Cytokine Milieu Supports *De Novo* Differentiation of IL-17-Producing T Cells. Immunity (2006) 24(2):179–89. 10.1016/j.immuni.2006.01.001 16473830

[12] Mucida D Park Y Kim G Turovskaya O Scott I Kronenberg M. Reciprocal TH17 and Regulatory T Cell Differentiation Mediated by Retinoic Acid. Science (2007) 317(5835):256–60. 10.1126/science.1145697 17569825

[13] Zhou L Lopes JE Chong MM Ivanov II Min R Victora GD. TGF-β-Induced Foxp3 Inhibits TH17 Cell Differentiation by Antagonizing Rorγt Function. Nature (2008) 453(7192):236–40. 10.1038/nature06878 PMC2597437 18368049

[14] Lozano-Ojalvo D Pérez-Rodríguez L Pablos-Tanarro A Molina E López-Fandiño R. Hydrolysed Ovalbumin Offers More Effective Preventive and Therapeutic Protection Against Egg Allergy Than the Intact Protein. Clin Exp Allergy (2017) 47(10):1342–54. 10.1111/cea.12989 28763132

[15] Fantini MC Becker C Monteleone G Pallone F Galle PR Neurath MF. TGF-Beta Induces a Regulatory Phenotype in CD4+CD25- T Cells Through Foxp3 Induction and Down-Regulation of Smad7. J Immunol (2004) 172(9):5149–53. 10.4049/jimmunol.172.9.5149 15100250

[16] Laurence A Tato CM Davidson TS Kanno Y Chen Z Yao Z. Interleukin-2 Signaling *via* STAT5 Constrains T Helper 17 Cell Generation. Immunity (2007) 26(3):371–81. 10.1016/j.immuni.2007.02.009 17363300

[17] Hill JA Hall JA Sun CM Cai Q Ghyselinck N Chambon P. Retinoic Acid Enhances Foxp3 Induction Indirectly by Relieving Inhibition From CD4+CD44hi Cells. Immunity (2008) 29(5):758–70. 10.1016/j.immuni.2008.09.018 PMC3140207 19006694

[18] Zhang X Chang Li X Xiao X Sun R Tian Z Wei H. CD4+CD62L+ Central Memory T Cells Can Be Converted to Foxp3+ T Cells. PloS One (2013) 8(10):e77322. 10.1371/journal.pone.007732 24155942 PMC3796486

[19] Ohnmacht C. Tolerance to the Intestinal Microbiota Mediated by ROR(γt) + Cells. Trends Immunol (2016) 37(7):477–86. 10.1016/j.it.2016.05.002 27255270

[20] Nolting J Daniel C Reuter S Stuelten C Li P Sucov H. Retinoic Acid can Enhance Conversion of Naive Into Regulatory T Cells Independently of Secreted Cytokines. J Exp Med (2009) 206(10):2131–9. 10.1084/jem.20090639 PMC2757891 19737861

[21] Baecher-Allan C Wolf E Hafler DA. Functional Analysis of Highly Defined, FACS-Isolated Populations of Human Regulatory CD4+CD25+ T Cells. Clin Immunol (2005) 115(1):10–8. 10.1016/j.clim.2005.02.018 15870015

[22] Ayyoub M Deknuydt F Raimbaud I Dousset C Leveque L Bioley G. Human Memory FOXP3+ Tregs Secrete IL-17 *Ex Vivo* and Constitutively Express the TH17 Lineage-Specific Transcription Factor RORgammat. Proc Natl Acad Sci USA (2009) 106(21):8635–40. 10.1073/pnas.0900621106 PMC2688993 19439651

[23] Voo KS Wang YH Santori FR Boggiano C Wang YH Arima K. Identification of IL-17-Producing FOXP3+ Regulatory T Cells in Humans. Proc Natl Acad Sci USA (2009) 106(12):4793–8. 10.1073/pnas.0900408106 PMC2653560 19273860

[24] McGeachy MJ Bak-Jensen KS Chen Y Tato CM Blumenschein W McClanahan T. TGF-β and IL-6 Drive the Production of IL-17 and IL-10 by T Cells and Restrain TH-17 Cell-Mediated Pathology. Nat Immunol (2007) 8(12):1390–7. 10.1038/ni1539 17994024

