# Supplementary material for: Retinoic Acid Induces Functionally Suppressive Foxp3+RORγt+ T Cells In Vitro
Source: Front Immunol. 2021 Aug 10;12:675733. doi: 10.3389/fimmu.2021.675733 (PMC8382797; doi:10.3389/fimmu.2021.675733)
Supplement: Supplementary file 1 [file Presentation_1.pptx]

## Slide 1
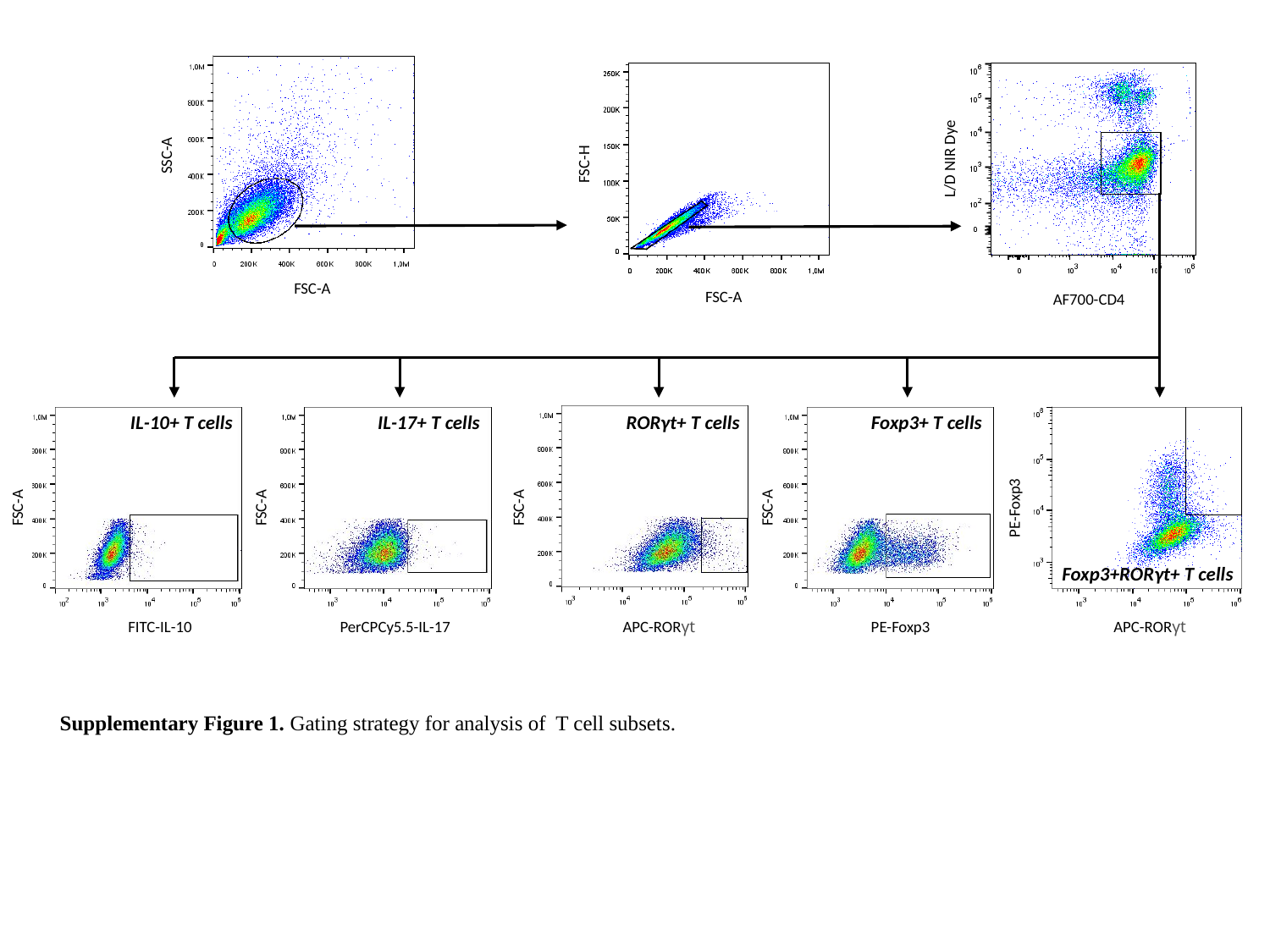

SSC-A
L/D NIR Dye
FSC-H
FSC-A
FSC-A
AF700-CD4
IL-10+ T cells
IL-17+ T cells
RORγt+ T cells
Foxp3+ T cells
FSC-A
FSC-A
FSC-A
FSC-A
PE-Foxp3
Foxp3+RORγt+ T cells
FITC-IL-10
PerCPCy5.5-IL-17
APC-RORγt
PE-Foxp3
APC-RORγt
Supplementary Figure 1. Gating strategy for analysis of T cell subsets.
