# Supplementary material for: Retinoic Acid Induces Functionally Suppressive Foxp3+RORγt+ T Cells In Vitro
Source: Front Immunol. 2021 Aug 10;12:675733. doi: 10.3389/fimmu.2021.675733 (PMC8382797; doi:10.3389/fimmu.2021.675733)
Supplement: Supplementary file 2 [file Table_1.docx]

**SUPPORTING INFORMATION**

**Table S1.** Primer sequences and conditions used in analyses of gene expression by qPCR.

| **Gene** | **Primer pairs** | **Reference** | **Cycling conditions** |
| --- | --- | --- | --- |
| *Actb* | *fw 5'* AGCTGCGTTTTACACCCTTT *3'*  *rv 5'* AAGCCATGCCAATGTTGTCT *3'* | [1] | *Pre-Incubation*  2 min 50ºC  *Incubation*  10 min 95ºC  **40 cycles**:  *Denaturation*  15 s 95ºC  *Annealing/Extension*  60 s 60ºC |
| *Foxp3* | *fw 5'*ACAACCTGAGCCTGCACAAGT *3'*  *rv 5'* GCCCACCTTTTCTTGGTTTTG *3'* | [1] |  |
| *Il17* | *fw 5'* TGCCTGTGGCACTGAAGTAG *3'*  *rv 5'* TTCATGGCTGCAGTGAAAAG *3'* | [2] |  |
| *Rorc* | *fw5'* TCACCTGTGAGGGGTGCAAG *3'*  *rv 5'* GTTCGGTCAATGGGGCAGTT *3'* | [3] |  |
| *Actb* | *fw 5'* AGCTGCGTTTTACACCCTTT *3'*  *rv 5'* AAGCCATGCCAATGTTGTCT *3'* | [1] | *Pre-Incubation*  2 min 50ºC  *Incubation*  10 min 95ºC  **40 cycles:**  *Denaturation*  15 s 95ºC  *Annealing/Extension*  45 s 58ºC + 15 s 60ºC |
| *Il6* | *fw 5'* TTCCATCCAGTTGCCTTCTTG *3'*  *rv 5'* GGGAGTGGTATCCTCTGTGAAGTC *3'* | [4] |  |
| *Il10* | *fw 5'* GCCTTATCGGAAATGATCCA *3'*  *rv 5'* AGGGGAGAAATCGATGACAG *3'* | [5] |  |
| *Tfgb1* | *fw* 5' TTGCTTCAGCTCCACAGAGA 3'  *rv* 5' TACTGTGTGTCCAGGCTCCA 3' | [5] |  |

*fw*, forward; *rv*, reverse

1. Cardoso, C.R., Provinciatto, P.R., Godoi, D.F., Ferreira, B.R., Teixeira, G., Rossi, M.A. (2009) IL-4 regulates susceptibility to intestinal inflammation in murine food allergy. *Am J Physiol Gastrointest Liver Physiol.* *296*, 593-600.
2. Lenoir, M., Del Carmen, S., Cortes-Perez, N.G., Lozano-Ojalvo, D., Muñoz-Provencio, D., Chain, F., Langella, P., de Moreno de LeBlanc, A., LeBlanc, J.G., Bermúdez-Humarán, L.G. (2016) Lactobacillus casei BL23 regulates Treg and Th17 T-cell populations and reduces DMH-associated colorectal cancer. *J Gastroenterol.* *51*, 862-873.
3. Yoh, K., Morito, N., Ojima, M., Shibuya, K., Yamashita, Y., Morishima, Y., Ishii, Y., Kusakabe, M., Nishikii, H., Fujita, A., Matsunaga, E., Okamura, M., Hamada, M., Suto, A., Nakajima, H., Shibuya, A., Yamagata, K., Takahashi, S. (2012) Overexpression of RORγt under control of the CD2 promoter induces polyclonal plasmacytosis and autoantibody production in transgenic mice. *Eur J Immunol.* *42*; 1999-2009.
4. Tordesillas, L., Goswami, R., Benedé, S., Grishina, G., Dunkin, D., Järvinen, K.M., Maleki, S.J., Sampson, H.A., Berin, M.C. (2014) Skin exposure promotes a Th2-dependent sensitization to peanut allergens. *J Clin Invest. 124*, 4965-4975.
5. Yang, M., Yang, C., Nau, F., Pasco, M., Juneja, L.R., Okubo, T., Mine, Y. (2009) Immunomodulatory effects of egg white enzymatic hydrolysates containing immunodominant epitopes in a Balb/c mouse model of egg allergy. *J Agric Food Chem.* *57*, 2241-2248.
